# Supplementary material for: Mathematical Modeling of ROS Production and Diode-like Behavior in the SDHA/SDHB Subcomplex of Succinate Dehydrogenases in Reverse Quinol-Fumarate Reductase Direction
Source: Int J Mol Sci. 2022 Dec 9;23(24):15596. doi: 10.3390/ijms232415596 (PMC9778801; doi:10.3390/ijms232415596)
Supplement: Supplementary file 1 [file ijms-23-15596-s001.zip › ijms-1956004-supplementary.pdf]

Supplementary Materials for

Mathematical Modeling of ROS Production and Diode-like Behavior in the SDHA/SDHB Subcomplex of Succinate Dehydrogenases in Reverse Quinol-Fumarate Reductase Direction

Nikolay I. Markevich [1,*] and Lubov N. Markevich [2]

[1] Institute of Theoretical and Experimental Biophysics of RAS, 142290 Pushchino, Russia
[2] Institute of Cell Biophysics of RAS, 142290 Pushchino, Russia
* Correspondence: markevich.nick@gmail.com

*Corresponding author.

Present address for correspondence:

Nikolay I. Markevich

Institute of Theoretical and Experimental Biophysics, Russian Academy of Sciences,

3 Institutskaya street,

Pushchino, Moscow region 14290, Russia

Email: markevich.nick@gmail.com

Phone: +7−9160983229

Mathematical model.

A computational model corresponding to the kinetic schemes in Fig.1 and Tables 1 and 2 in the main text consists of 13 ordinary differential equations (ODE) and 4 moiety conservation equations. The model was implemented in DBSolve Optimum software available at website **http://insysbio.ru.**

Additionally, the model is presented in SBML format by separate file: **Markevich_Final Reverse Scheme SDHA-B.xml** as Supporting information. It should point out that expressions for $d(O_2^{\cdot-})/dt$ and $d(H_2O_2)/dt$ after recover the model from SBML format have to take into account the ratio Wimb/Wmx where Wimb and Wmx the fractional volume ratio of the inner membrane and matrix, respectively, to the total mitochondrial volume. See below *The system of ODE.*

The system of ODE. The system of ODE that was analyzed computationally in the present study can be written as follows:

$d([3Fe\text{-}4S])/dt = 2 * (-V_1 + V_2) + V_{20};$

$d([4Fe\text{-}4S])/dt = 2 * (-V_2 + V_3);$

$d([2Fe\text{-}2S])/dt = - 2 * V_3 + V_5 + V_6 + V_9 + V_{11} + V_{13} + V_{15};$

$d(O_2^{\cdot-})/dt = Wimb/Wmx * (V_{18} + V_{19} + V_{20}) - V_{21}*2;$

$d(H_2O_2)/dt = Wimb/Wmx * V_{17} + V_{21} - V_{22};$

$d(FAD)/dt = - V_4 + V_8 - V_9 + V_{17} + V_{19};$

$d(FAD\_fum)/dt = V_4 - V_5;$ (1)

$d(FADH^{\cdot}\_fum)/dt = V_5 - V_6 + V_{10};$

$d(FADH2\_fum)/dt = V_6 - V_7 + V_{12};$

$d(FAD\_suc)/dt = V_7 - V_8 - V_{13};$

$d(FADH^{\cdot}\_suc)/dt = V_{13} - V_{14} - V_{15};$

$d(FADH^{\cdot})/dt = V_9 - V_{10} - V_{11} + V_{14} + V_{18} - V_{19};$

$d(FADH2)/dt = V_{11} - V_{12} + V_{16} - V_{17} - V_{18};$

Where, the left-hand sides of the equations contain time derivatives of the concentration of various redox centers in the oxidized or reduced states, and the right-hand sides of the equations represent the rates V_i (i=1, 22) of change of these variables due to electron transfer or in the process of binding/dissociation with the corresponding centers of SDH. The expressions for rates V_i in SDH are presented in Table 1, and all the parameters are in Table 2 in the main text. Here, the fractional volume ratio of matrix, Vmx (Wmx=Vmx/Vmit) and inner membrane, Vimb (Wimb=Vimb/Vmit) to the total mitochondrial volume Vmit equal approximately 0.24 and 0.652, respectively.

*Conserved moieties (in μM).*The model took into account the laws of conservation of the total concentration in the membrane of both SDH and the pools of different redox centers.

The total concentration of FAD centers at different states in SDH is:

$Pool[1] = FAD + FAD\_suc + FAD\_fum + FADH^{\cdot} + FADH^{\cdot}\_suc + FADH^{\cdot}\_fum + FADH2 + FADH2\_suc + FADH2\_fum;$

The total concentration of [2Fe–2S] clusters at different oxidized and reduced states in SDH:

$Pool[2] = [2Fe–2S]^{-} + [2Fe–2S];$

The total concentration of [4Fe–4S] clusters at different oxidized and reduced states in SDH:

$Pool[3] = [4Fe–4S]^{-} + [4Fe–4S];$

The total concentration of [3Fe–4S] clusters at different oxidized and reduced states in SDH:

$Pool[4] = [3Fe–4S]^{-} + [3Fe–4S];$

The concentration of pools of different redox centers in SDH is taken to be equal to the total concentration of SDH. It is taken that Pool[1] = Pool[2] = Pool[3] = Pool[4] = 235 μM.

Expressions for dependent variables. The expressions for the concentration of all 4 dependent variables used in ODE are easily calculated from the pools of different variables and are presented below.

$FADH2\_suc=-(FAD+FAD\_suc+FAD\_fum + FADH^{\cdot} + FADH^{\cdot}\_suc +FADH^{\cdot}\_fum +FADH2 + FADH2\_fum)+Pool[1];$

$[2Fe–2S]^{-} = - [2Fe–2S] + Pool[2];$

$[4Fe–4S]^{-} = - [4Fe–4S] + Pool[3];$

$[3Fe–4S]^{-} = - [3Fe–4S] + Pool[4];$

Explicit functions. Expressions for some important functions used in computations are presented below.

$VO_2^{\cdot -} = V_{18} + V_{19} + V_{20}$; the total rate of $O_2^{\cdot -}$ production by the subcomplex SDHA/SDHB of SDH in μM/s.

$VH_2O_2 = V_{17} * Wimb/Wmx + V_{21}$; the total rate of H_2O_2 production by the subcomplex SDHA/SDHB of SDH in μM/s expressed as WHOLE MITO Rates (Wimb=Vimb/Vmit, where Vimb and Vmit are volumes of the inner membrane and whole mitochondria, respectively) and Wimb=0.24.

It should be pointed that the stationary rate $VH_2O_2 = V_{22}$, as follows from the system of ODE (1).

Dimension of local and whole mitochondrial concentration and rates. This paragraph is described in detail in our previous work [1]. Experimental data on intramembrane protein concentrations presented in Table 2 are usually presented in nmole/mg mitochondrial protein, whereas we use concentration units (μM) in our computational model. Moreover, we use in the model local concentrations of proteins in different compartments of mitochondria, normalized by the relative volume fractions of these compartments. Therefore, matrix superoxide $O_2^{\cdot -}$ and H_2O_2

concentrations were normalized by the matrix water volume (V_{MX}) and concentrations of all intramembrane proteins of the SDHA/SDHB subunits of SDH were normalized by the inner membrane volume (V_{IMB}). First, we normalized the concentration of all proteins by the total mitochondrial volume, V_{MIT}; then, total mitochondrial concentrations were translated into local concentrations using the water space fraction of matrix ($W_{MX}=V_{MX}/V_{MIT}$) and IMS ($W_{IMS}=V_{IMS}/V_{MIT}$), and the fractional volume ratio of inner membrane ($W_{IMB}=V_{IMB}/V_{MIT}$) to the total mitochondrial volume. In order to calculate W_{MX}, W_{IMS} and the total mitochondrial water space fraction, $W_{MITW}=V_{MITW}/V_{MIT}$, where V_{MITW} is the total mitochondrial water volume, we used the following experimental data. The mitochondrial water weight fraction, m_w/m_{mit}, where m_w and m_{mit} is the mass of mitochondrial water and mitochondria, respectively, equals to 0.664 g/g wet weight for a total mitochondrial density, ρ_{mit}, ($\rho_{mit} = m_{mit} / V_{MIT}$) of 1.09 g/ml [2]. Because $m_w = \rho_w \cdot V_{MITW}$ and $m_{mit} = \rho_{mit} \cdot V_{MIT}$, where the water densities, ρ_w, and ρ_{mit} are 1 and 1.09 g/ml, respectively, we can calculate the total mitochondrial water space fraction, W_{MITW}. Since $m_w/m_{mit} = \rho_w \cdot V_{MITW} / \rho_{mit} \cdot V_{MIT} = W_{MITW} \cdot 1$ g/ml / 1.09 g/ml =0.664 g/g, so the total mitochondrial water space fraction, W_{MITW}, is $0.664 \cdot 1.09=0.724$.

Taking into account that $W_{IMS} \approx 1/14 \approx 0.07$ of the total mitochondrial water space for the orthodox configuration [3], the matrix water space fraction $W_{MX} = W_{MITW} - W_{IMS} = 0.652$. These values of W_{IMS} and W_{MX} are in agreement with those used in [4]. The value of W_{IMB} was calculated as follows: The volume and inner membrane surface area of an average rat liver mitochondrion are 0.27 μm^3 and 6.47 μm^2, respectively [5]. Assuming an average inner membrane thickness of about 0.01 μm, $V_{IMB}=6.47\ \mu m^2 \cdot 0.01\ \mu m = 0.0647\ \mu m^3$ and the inner membrane space fraction ($W_{IMB}=V_{IMB}/V_{MIT}$) of a mitochondrion is approximately 0.24.

The mitochondrial protein weight fraction W_{wprot} is about 0.25 g/g wet weight [2], i.e., 1 mg mitochondrial protein corresponds to 4 mg mitochondrial wet weight and occupies 4mg/1090 mg/ml = 3.67·μl. Therefore, a mitochondrial content of any metabolite of 1 nmol/mg mitochondrial protein, when normalized to total mitochondrial volume, is equal to a concentration of 10^{-9} mole/ $3.67 \cdot 10^{-6}$ l = 273 μM, i.e. 1 μM = 3.67 pmol/mg mitochondrial protein.

In order to present computer simulated rates of respiration and ROS production, which occur only in the inner membrane, in units of whole mitochondrial rates we multiplied all the rates of intramembrane processes by $W_{IMB} = 0.24$.

In addition, in order to compare computer simulated rates of respiration and ROS production presented in the current paper in μM/s with experimentally observed rates expressed in pmol/min/mg protein the computer simulated rates can be multiplied by a factor of 3.67*60 = 220, i.e. 1 μM/s = 220 pmol/min/ mg mitochondrial protein.

References

1. Markevich N.I., Hoek J.B. Computational modeling analysis of mitochondrial superoxide production under varying substrate conditions and upon inhibition of different segments of the electron transport chain. *Biochim. Biophys. Acta*. 2015;1847:656-679. doi: 10.1016/j.bbabio.2015.04.005
2. Vinnakota K.C., Bassingthwaighte J.B. Myocardial density and composition: a basis for calculating intracellular metabolite concentrations. *Am. J. Physiol. Heart Circ. Physiol.* 2004;286:H1742-1749. doi: 10.1152/ajpheart.00478.2003
3. Hackenbrock C.R., Chazotte B., Gupte S.S. The random collision model and a critical assessment of diffusion and collision in mitochondrial electron transport. *J. Bioenerg. Biomembr*. 1986;18:331-368. doi: 10.1007/BF00743010
4. Wu F., Yang F., Vinnakota K.C., Beard D.A. Computer modeling of mitochondrial tricarboxylic acid cycle, oxidative phosphorylation, metabolite transport, and electrophysiology. *J. Biol. Chem*. 2007;282:24525-24537. doi: 10.1074/jbc.M701024200
5. Schwerzmann K., Cruz-Orive L.M., Eggman R., Sanger A., Weibel E.R. Molecular architecture of the inner membrane of mitochondria from rat liver: a combined biochemical and stereological study. *J. Cell. Biol*. 1986;102:97-103. doi: 10.1083/jcb.102.1.97
